# Hyperthyroidism in non-seminomatous testicular germ cell tumors: two case reports and literature review

**DOI:** 10.3389/fonc.2024.1338438

**Published:** 2024-03-27

**Authors:** Diletta Favero, Christoph Oing, Christoph Seidel, Pasquale Rescigno, Fabio Catalano, Malvina Cremante, Sara Elena Rebuzzi, Federico Gatto, Giovanni Rosti, Diego Ferone, Giuseppe Fornarini, Francesco Cocchiara

**Affiliations:** ^1^ Department of Internal Medicine and Medical Specialties (Di.M.I.), School of Medicine, University of Genova, Genova, Italy; ^2^ Department of Medical Oncology, U.O. Clinica di Oncologia Medica, Istituto di Ricovero e Cura a Carattere Scientifico (IRCCS) Ospedale Policlinico San Martino, Genova, Italy; ^3^ Translational and Clinical Research Institute, Centre for Cancer, Newcastle University, Newcastle upon Tyne, United Kingdom; ^4^ Mildred Scheel Cancer Career Centre HaTriCS4, University Cancer Centre Hamburg, University Medical Centre Eppendorf, Hamburg, Germany; ^5^ Department of Oncology, Hematology and Bone Marrow Transplantation with Division of Pneumology, University Medical Centre Eppendorf, Hamburg, Germany; ^6^ Candiolo Cancer Institute Fondazione del Piemonte per l’Oncologia-Istituto di Ricovero e Cura a Carattere Scientifico (FPO-IRCCS), Candiolo, Italy; ^7^ Medical Oncology Unit 1, Istituto di Ricovero e Cura a Carattere Scientifico (IRCCS) Ospedale Policlinico San Martino, Genova, Italy; ^8^ Medical Oncology Unit, Ospedale San Paolo, Savona, Italy; ^9^ Department of Internal Medicine and Medical Specialties (Di.M.I.), University of Genova, Genova, Italy; ^10^ Endocrinology Unit, Department of Internal Medicine, Istituto di Ricovero e Cura a Carattere Scientifico (IRCCS) Ospedale Policlinico San Martino, Genova, Italy; ^11^ Oncology Department, Fondazione Istituto di Ricovero e Cura a Carattere Scientifico (IRCCS) Policlinico San Matteo, Pavia, Italy; ^12^ Endocrinology Unit, Department of Internal Medicine and Medical Specialties (Di.M.I.), Istituto di Ricovero e Cura a Carattere Scientifico (IRCCS) Ospedale Policlinico San Martino, University of Genova, Genova, Italy

**Keywords:** non-seminomatous testicular germ cell tumors, thyrotoxicosis, hyperthyroidism, human chorionic gonadotropin, TSH receptor

## Abstract

**Background:**

Human chorionic gonadotropin (hCG)–induced hyperthyroidism is a rare paraneoplastic syndrome observed in non-seminomatous testicular germ cell tumors, due to a cross-reaction between the β-subunit of hCG with the thyroid-stimulating hormone receptor. The precise prevalence of this paraneoplastic phenomenon is unclear as, in the majority of cases, hyperthyroidism remains subclinical.

**Case presentation:**

Here, we present two cases of advanced metastatic non-seminomatous testicular germ cell tumors where patients exhibited signs and symptoms of thyrotoxicosis at primary diagnosis due to excessive serum β-hCG elevation, with complete remission of symptomatology after the start of oncological treatments and no signs of relapse at the time of publication of this report. Additionally, we provide a comprehensive review of the existing literature concerning this uncommon occurrence.

**Conclusion:**

Despite being a rare event, the presence of hyperthyroidism or thyrotoxicosis without clear etiology in a young man should lead to consider less frequent causes such as testicular tumors. Even if patients typically have mild symptoms that resolve after chemotherapy, in rare cases, it can be a life-threatening condition that requires prompt recognition and specific intervention.

## Introduction

The term “thyrotoxicosis” refers to a clinical condition that results from elevated circulating thyroid hormone action within the body. The main causes include Graves’ disease, toxic adenoma, toxic multinodular goitre and thyroiditis, as well as hyperthyroidism induced by iodinated contrast media or amiodarone. Rarely, thyrotoxicosis may also be related to a paraneoplastic syndrome (1–3).

Human chorionic gonadotropin (hCG)–induced hyperthyroidism represents a rare paraneoplastic syndrome in non-seminomatous testicular germ cell tumors (NSTGCTs), in which the excessive secretion of the β-subunit of hCG (β-hCG) results in hyperthyroidism through a cross-reaction with the thyroid-stimulating hormone (TSH) receptor (4). Excessive β-hCG levels can be present in NSTGCTs with a predominant choriocarcinoma component (5). The exact prevalence of this paraneoplastic phenomenon is unknown: in most cases, hyperthyroidism remains subclinical, and, thus, only a limited number of cases with clinically symptomatic hyperthyroidism have been reported in the literature.

Here, we present two cases of advanced NSTGCT with signs and symptoms of thyrotoxicosis at primary diagnosis due to excessive serum β-hCG elevations and a review of the available literature on this rare phenomenon.

## Case presentation I

A previously healthy 38-year-old man presented to the Emergency Department of the “San Martino” University Hospital of Genoa with classic symptoms of thyrotoxicosis: tachycardia with palpitations, insomnia, restlessness, tremors in the limbs, loss of appetite, and weight loss of about 5 kg.

His thyroid function was tested, and the results validated the diagnosis of thyrotoxicosis [TSH suppressed and free T4 (fT4), 52.7 pmol/L; upper limit of normal (ULN), 18 pmol/L]. The remaining blood tests were normal, except for a mild anemia [hemoglobin (Hgb), 124 g/L; normal range, 140–180 g/L] and a slight elevation of Gamma-glutamyl transferase (GGT, 83 IU/L; ULN, 49 IU/L).

Clinically, a thyroid gland of increased volume was detected on cervical palpation but had no exophthalmos and no cervical lymphadenopathy.

A thyroid ultrasound (US) showed an enlarged and heterogeneous thyroid gland with hypervascular flow on Doppler US.

Further diagnostic work-up for hyperthyroidism including autoimmune antibodies as well as iodiuria yielded negative results.

Treatment with methimazole of 5 mg six times a day and propranolol of 20 mg twice a day was initiated, and the patients was discharged with close endocrinological follow-up.

However, shortly after, the patient was hospitalized because of worsening of the clinical condition with dyspnea and intense asthenia.

At admission, he was tachycardic, tachypneic, and fatigued. The vital signs were unremarkable, including an oxygen saturation of 99% on room air. Abnormal findings on physical examination included fine crepitations above both lungs and a significantly enlarged left testicle.

Initial laboratory investigation revealed microcytic anemia (Hgb, 91 g/L), excessively high serum β-hCG of >1 × 10^6^ IU/L (ULN < 3 IU/L), α-fetoprotein (AFP) of 846 ng/mL (ULN < 13.6 ng/mL), and an elevated lactate dehydrogenase (LDH) of 981 U/L (normal range, 135–250 IU/L).

Despite the ongoing thyrostatic therapy, thyroid function tests revealed elevated serum fT3 and fT4 levels, along with a suppressed TSH level. Laboratory test results and markers trend are summarized in **Table 1**.

**Table 1:** Case 1: Thyroid function assessment and markers trend.

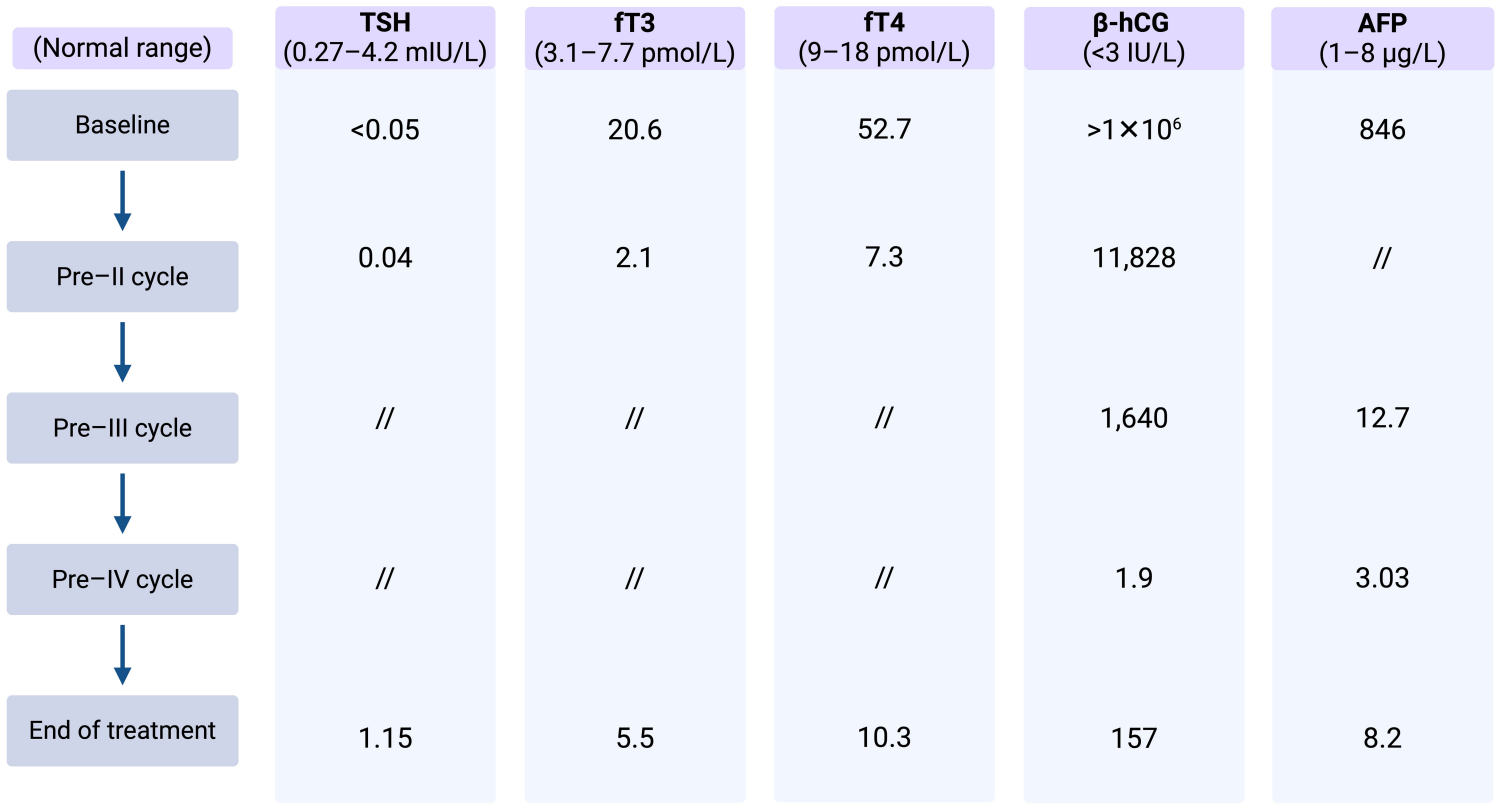

A testicular US revealed a heterogeneous left intratesticular mass of 9 cm × 10 cm × 11 cm in size, suspicious of a testicular primary tumor. A computed tomography (CT) scan of the head, chest, and abdomen showed diffuse metastases in the lungs, liver, bone, and retroperitoneal lymph nodes.

Clinical diagnosis of metastatic NSTGCT was made on the basis of the findings of a solid testicular mass with multiple metastases and very high levels of serum β-hCG, and the thyrotoxicosis was attributed to be a paraneoplastic phenomenon.

The patient underwent immediate left radical inguinal orchiectomy, which confirmed the diagnosis of an embryonal carcinoma of the testis, pT2 cN3 cM1b S3, Union for International Cancer Control (UICC) clinical stage IIIC (6).

He was classified as having a poor prognosis in view of his high level of β-hCG and metastatic sites, based on the International Germ Cell Cancer Collaborative Group (IGCCCG) criteria, and was started on standard conventional chemotherapy with cisplatin 20 mg/m² and etoposide 100 mg/m^2^ on days 1–5 and bleomycin of 30 mg on days 2, 9, and 16 Platinol, Etoposide and Bleomycin (PEB) for four treatment cycles (7).

After completing his first chemotherapy cycle, his serum β-hCG decreased significantly, and the thyroid function improved to near normalization (**Table 1**) with complete resolution of his symptoms. Methimazole was decreased from 30 mg to 10 mg daily and was discontinued at the start of the second cycle of chemotherapy.

After three cycles of treatment, his β-hCG plummeted to 1.9 IU/L but started to rise again subsequently in keeping with acquired cisplatin resistance.

At the end of the four cycles of therapy, a re-evaluation CT scan was performed with evidence of a marker-positive partial remission of all metastatic sites.

After 3 months, β-hCG increased to 310 IU/L, whereas the thyroid function remained normal. Consequently, the patient underwent salvage chemotherapy with one cycle of induction with paclitaxel of 250 mg/m^2^ on day 1 and ifosfamide of 3,000 mg/m^2^ on days 2–3 (TI) for stem cell mobilization and subsequent high-dose carboplatin Area Under the Curve (AUC) of 7 and etoposide of 400 mg/m^2^ on days 1–3 for three treatment cycles with autologous bone marrow stem cell support.

At the end of the high-dose chemotherapy, the patient’s β-hCG level decreased to 8 IU/L, and the CT scan still showed residual lesions in all metastatic sites.

After multidisciplinary discussion, it was decided to proceed with debulking surgery of the residual hepatic and retroperitoneal lesions with no viable germ cell tumor cells according to histopathology. Two irresectable liver lesions were additionally treated with radiofrequency ablation.

The patient was also started on maintenance oral therapy with VP100, which was suspended because of poor tolerance. Maintenance oral etoposide after salvage therapy is not a standard practice in Italy but retrospective data has hinted at its potential benefits (8), and the results of an ongoing randomized clinical trial are pending and will provide valuable insights (9).

At the last re-evaluation, β-hCG was stable at 8 IU/L, whereas the CT scan showed stable pulmonary micronodules and signs of hepatic metastasectomy and lymphadenectomy. To date, three years after completing the salvage treatment, the patient remains relapse-free and euthyroid, with ongoing regular intensive follow-up assessments.

## Case presentation II

A previously healthy 18-year-old man presented at the Emergency Medicine Department of the University Medical Centre Hamburg-Eppendorf with quickly worsening shortness of breath, fatigue, polyuria, polydipsia, and unintended weight loss. Vital signs were abnormal with tachycardia and an oxygen saturation of 88% on room air.

Physical examination revealed reduced breathing sounds over both lungs and a slightly enlarged left testicle.

**Figure 1:**
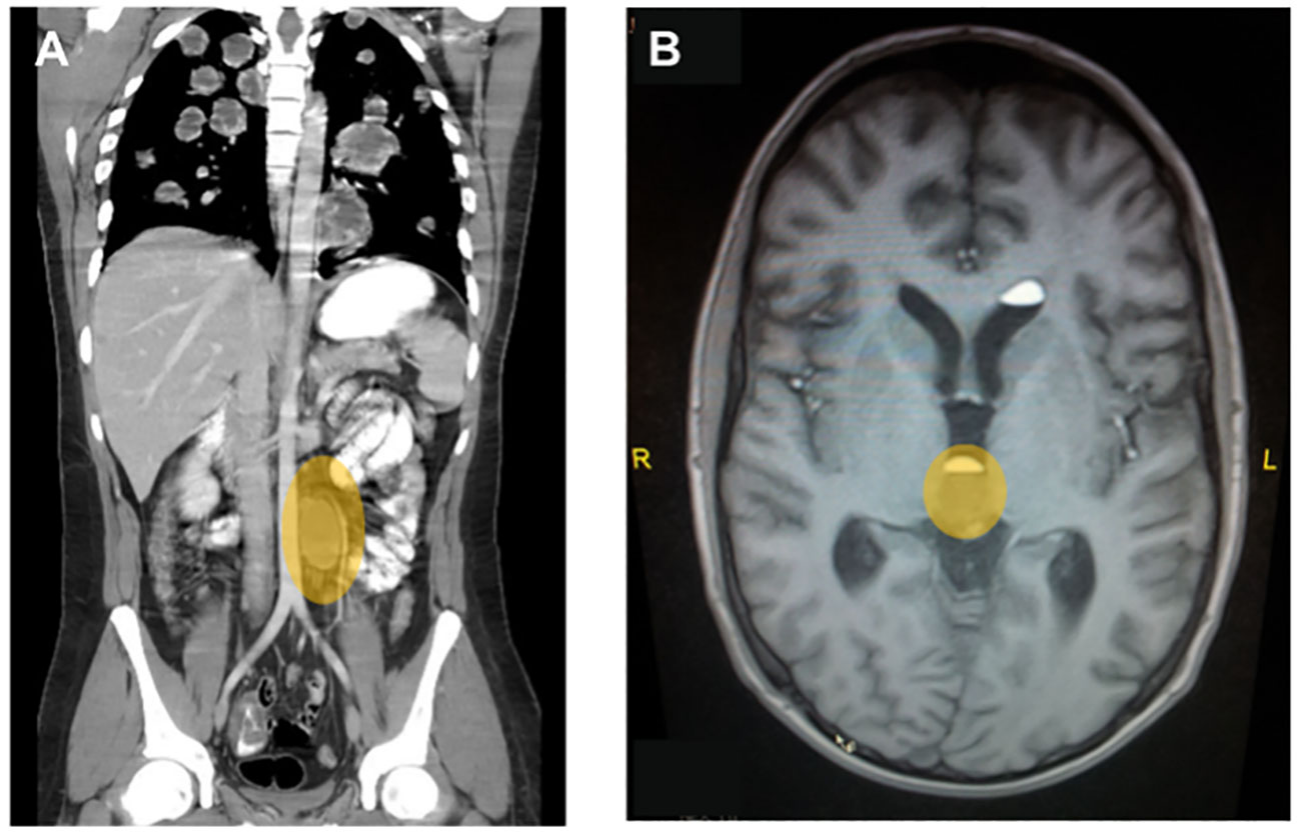
Representative diagnostic images at primary diagnosis for case 2. **(A)** Contrast-enhanced computed tomography of chest abdomen and pelvis showing multiple bilateral, centrally necrotic lung metastases and a singular retroperitoneal para-aortic lymph node metastasis. **(B)** T2-weighted image of a whole-brain MRI showing a central metastasis close to the pituitary gland, which lead to a clinically significant diabetes mellitus with polyuria and polydipsia.

A chest X-ray showed multiple bi-pulmonary nodules mainly in the inferior lobes and the right middle lobe. A subsequent CT of thorax, abdomen, and pelvis confirmed the widespread pulmonary metastatic lesions and also showed a large centrally necrotic left-sided retroperitoneal lymph node of 5.3 cm × 4.6 cm adjacent to the pancreatic tail (**Figure 1**).

Thyroid function assessment prior to CT scanning revealed a suppressed TSH and a significantly elevated serum level of free T4 in line with thyrotoxicosis. Alongside, germ cell tumor serum tumor markers were found to be elevated with a massively elevated β-hCG of 2.39 × 10^6^ IU/L (ULN < 3 IU/L), mildly elevated AFP at 21.3 µg/L (ULN, 8 µg/L), and an LDH of 835 IU/L (ULN, 250 IU/L). Laboratory test results and markers trend are summarized in **Table 2**.

**Table 2:** Case 2: Thyroid function assessment and markers trend.

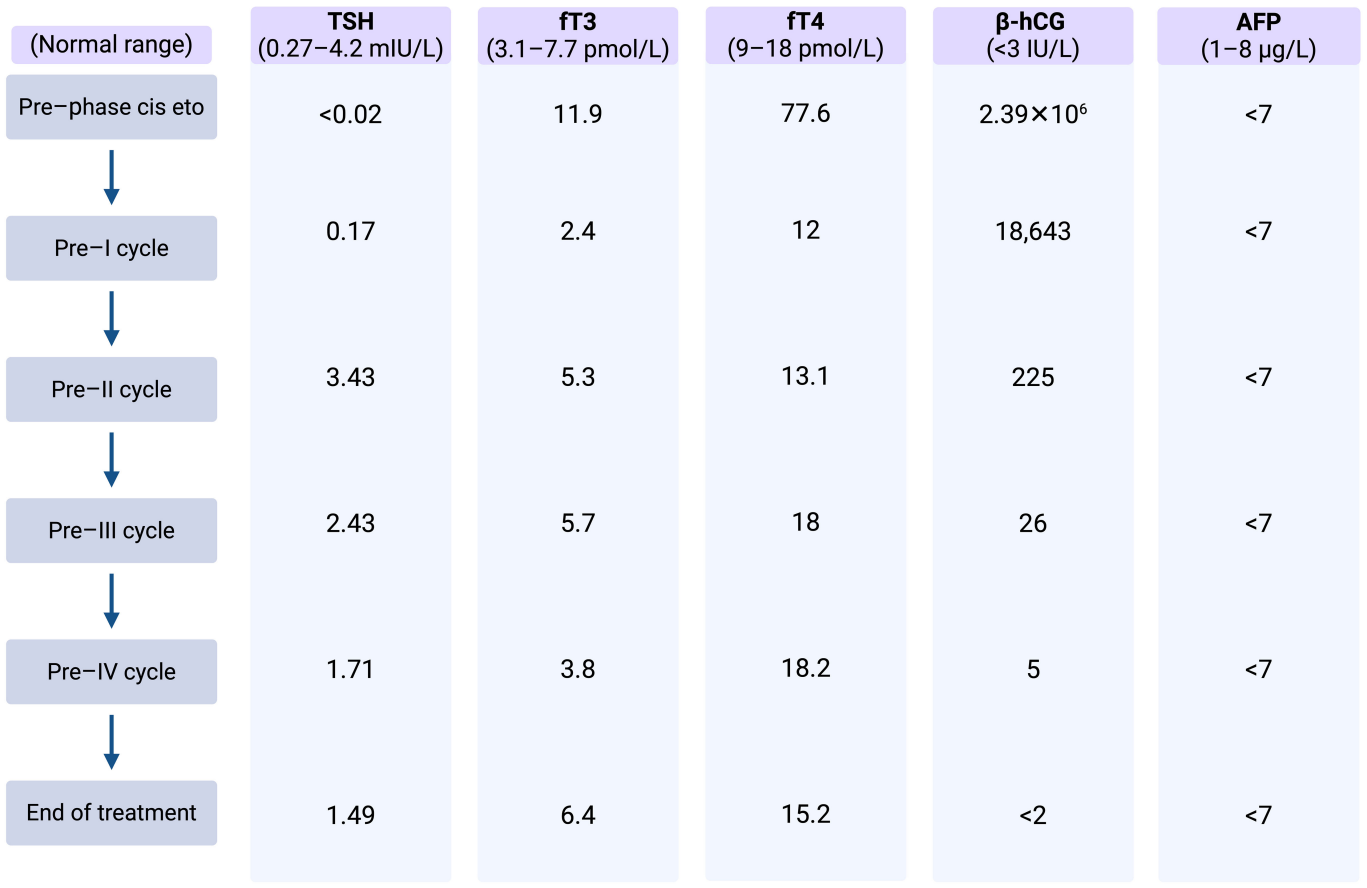

An additional testicular US showed a large, hypovascularized lesion of 1.5 cm × 1.3 cm in the left testicle suspicious for a germ cell tumor.

Based on the clinical and laboratory findings, this patient was diagnosed with a metastatic non-seminoma, TNM stage cTx N3 M1b S3, clinical stage IIIC with poor prognosis according to the IGCCCG risk criteria (6, 7).

In light of the excessive β-hCG elevation and the extensive pulmonary metastases despite limited lymphonodal spread, it was assumed to be a predominant choriocarcinoma. Consequently, the patient underwent an additional magnetic resonance imaging (MRI) of the brain, which revealed metastases in the left hemisphere with perifocal edema, in the right splenium and at the base of the pituitary gland (**Figure 1**), the latter causing an additionally diagnosed diabetes insipidus as an explanation for his polydipsia and polyuria of about 10 L/day.

Because of the imminent respiratory failure and thyrotoxicosis, the patient was transferred to the intensive care unit and immediately commenced cytotoxic chemotherapy without primary orchiectomy.

Given the high risk of treatment-induced tumor lysis syndrome, the patient initially received one cycle of cytoreductive chemotherapy with cisplatin of 20 mg/m^2^ and etoposide of 75 mg/m^2^ on days 1–3, which lead to a major pulmonary bleeding and a pneumothorax, which required invasive ventilation due to global respiratory failure in terms of an acute respiratory distress syndrome showing the full clinical picture of a choriocarcinoma syndrome.

Following the diagnosis, in addition, carbimazole and propranolol were administered to manage the thyrotoxicosis, and nasal vasopressin was prescribed for the diabetes insipidus.

Upon clinical stabilization and extubation, first-line systemic therapy with cisplatin of 20 mg/m^2^, etoposide of 75 mg/m^2^, and ifosfamide of 1.2 g/m^2^ on days 1–5 etoposide (VePesid), ifosfamide and cisplatin (Platinol) (VIP) was given followed by successful autologous stem cell harvest of 3 × 6.3 × 10^6^ CD34+ cells.

The patient was transferred to the Oncology Department and treated with three sequential cycles of high-dose VIP (HD-VIP; cisplatin of 20 mg/m^2^, etoposide of 300 mg/m^2^, and ifosfamide of 2.4 g/m^2^ on days 1–5) with autologous stem cell support on day 7.

The β-hCG values dropped continuously during the chemotherapy and reached normal range after the third cycle of HD-VIP achieving a marker-negative partial remission with residual masses in the retroperitoneum and lungs and equivocal changes on whole-brain MRI. The thyroid function parameters normalized quickly along the dropping β-hCG, but the patient still depended on desmopressin substitution to control the diabetes insipidus.

Six weeks after the completion of chemotherapy with continued normalized serum tumor markers, the patient underwent retroperitoneal residual mass resection and left-sided orchiectomy showing necrosis only in the resected specimens. Complete resection of the pulmonary masses was deemed unfeasible and thus omitted, whereas additional whole-brain radiotherapy (10 × 3 Gy fractions, cumulative dose 30 Gy) was applied to control the multifocal brain metastases.

After a follow-up of so far 10 years, the patient never relapsed and shows an ongoing marker-negative partial remission with small pulmonary nodules suspected as residual fibrotic residues.

Long-term sequelae of the intensified treatment include fatigue and an ongoing diabetes insipidus sufficiently controlled with transnasal desmopressin substitution.

## Discussion

Testicular germ cell tumors (TGCTs) are a relatively rare type of cancer, accounting for about 1% of all cancers in men. However, TGCTs are the most common solid tumor in men aged between 20 and 34 years (10).

Expression of hCG physiologically originates from syncytiotrophoblastic cells of the placenta during pregnancy. Because non-seminomatous germ cell tumors originate from totipotential embryonic germ cell capable of generating all embryological differentiation lineages, including somatic (teratoma) and extra-embryonic (choriocarcinoma and yolk sac tumor), syncytiotrophoblastic cells can also be found in NSTGCTs and less prominently also in seminomas (11). All patients with choriocarcinomas and 40% to 60% of patients with embryonal cell carcinomas display elevated serum levels of β-hCG, whereas approximately 10%–20% of patients with pure seminoma have elevated serum β-hCG levels, which rarely exceed 500 IU/L (5). In non-seminomas, β-hCG serum levels independently predict patient survival and represent a risk factor in the IGCCCG risk classification for metastatic NSTGCTs (12). Moreover, β-hCG serum levels of >50,000 IU/L together with widespread lung metastases are risk factors for the development of choriocarcinoma syndrome, a rare complication with signs of pulmonary hemorrhage and respiratory distress syndrome (13). Paraneoplastic hCG production has also been described in liver, pancreatic, gastric, breast, kidney, and bladder cancers (5).

Interestingly, high β-hCG serum levels are linked to hyperthyroidism. This is due to a cross-reaction of β-hCG with the TSH receptor, which is based on the structural similarity between the two glycoprotein hormones (hCG and TSH) and their receptors. This not only elucidates the pathophysiology of gestational thyrotoxicosis but also clarifies the underlying mechanism of paraneoplastic hyperthyroidism in men with TGCTs exhibiting elevated serum β-hCG levels, as well as in women with gestational trophoblastic disease (1, 14, 15). Cross-activation of the TSH receptor by β-hCG occurs only at markedly high serum levels, given that β-hCG acts as a relatively weak agonist of the TSH receptor (1).

Oosting et al. evaluated prospectively the thyroid function of 144 patients treated with chemotherapy for disseminated NSTGCTs. They identified five patients with hyperthyroidism. All five patients had β-hCG levels >50,000 IU/L corresponding to 50% of patients with such high β-hCG levels, whereas none of the other 134 patients with low or intermediate hCG levels (β-hCG <5,000 IU/L or >5,000 but <50,000 IU/L) had hyperthyroidism (4). Furthermore, the likelihood of a patient developing thyrotoxicosis is influenced not just by the quantity but also by the quality of hCG, as structural changes in hCG can result in enhanced thyrotropic activity. Specifically, variants of hCG produced paraneoplastically in patients with cancer often display excessive sialylation, and the extent of this sialylation correlates with the thyrotropic activity of the hCG (16). Additionally, other molecular variants that enhance the thyrotropic potency of hCG include truncated molecules that lack the carboxyl-terminal (C-terminal) tail. Indeed, the role of C-terminal peptide seems to be to prevent hyperthyroidism during the first trimester of pregnancy, when a large amount of hCG is produced by the placenta, and this also explains why the very high hCG levels in pregnancy are not generally associated with thyrotoxicosis (14).

Primary treatment of paraneoplastic hyperthyroidism is to appropriately treat the hCG-secreting tumor. Meanwhile, hyperthyroidism should be treated to control the negative systemic consequences of thyrotoxicosis. The pharmacological treatment can include anti-thyroid drugs, inorganic iodide, corticosteroids, β-blockers, and antipyretic agents depending on clinical presentation (1). Treatment should be given according to local policy with the help of endocrinology specialists. Both presented patients received therapy with anti-thyroid drugs and β-blockers to control the excessive thyroid function. The first patient was treated with methimazole of 30 mg/day and propranolol of 40 mg/day, and the other patient received carbimazol of 15 mg/day and propranolol of 40 mg/day. In patients with significantly symptomatic thyrotoxicosis, the consideration of β-blockers is advised, as β-adrenergic blocking agents can alleviate symptoms by inhibiting the effects of adrenaline on the body. Additionally, at higher doses, propranolol is known to suppress the conversion of thyroxine (T4) to the more active triiodothyronine (T3), offering a therapeutic advantage in the regulation of thyroid hormone levels in cases of thyrotoxicosis (1).

For patients with metastatic TGCT and a poor prognosis (based on the criteria of the international classification IGCCCG), surgery alone is insufficient, but cisplatin-based chemotherapy is effective to control the disease and thereby decrease β-hCG levels, which, in turn, reduces thyrotoxic symptoms, as reported for both cases (17). Certainly, thyrotoxicosis encompasses an additional thread and may contribute to a critical condition, as described for case II, but curative chemotherapy must not be postponed but started immediately along initiation of thyrostatic treatment.

As the prevalence of clinically symptomatic hyperthyroidism in men with β-hCG–secreting TGCTs is low, there are only a few cases described in literature (18, 19).

In 2000, Goodarzi and Van Herle published a review of the literature since 1964 describing 17 cases of male TGCT patients with signs and symptoms of thyrotoxicosis due to excessively elevated β-hCG levels. For two patients, survival information was not available; seven patients were alive at the time of the case report; and among the eight patients that had died, one had succumbed to thyroid storm, whereas the others died as a consequence of tumor progression (19).

We performed a literature search using the electronic databases PubMed, including case reports on patients with testicular neoplasms and hyperthyroidism or thyrotoxicosis published since 2000 and excluding non-English articles. We used the following search terms: “(hyperthyroidism) AND (germ-cell tumor),” “(hyperthyroidism) AND (germ-cell cancer),” and “(hyperthyroidism) AND “(human chorionic gonadotropin)”.

In **Table 3**, the 14 cases reported in literature that we found are summarized (18–31). Patients’ age at diagnosis, when reported, ranged from 18 to 58 years. Most patients (9/17) began with symptoms related to hyperthyroidism, whereas, in other patients, the neoplastic disease presented with different symptoms (e.g., testicular pain, abdominal pain, dysuria, or black stools) despite the presence of hyperthyroidism on laboratory tests. Only 10 authors reported the tumor histological subtype: in particular, we found eight cases of choriocarcinoma, two of which were part of a mixed tumor (yolk sac tumor and embryonal carcinoma or yolk sac tumor and teratoma); the remaining two patients had, respectively, an embryonal carcinoma and a mixed germ cell tumor (seminoma, embryonal cancer, and teratoma). All patients had very high levels of β-HCG, varying from 1,063 IU/L to 1,118,053 IU/L, and the majority was at a metastatic stage. At the time of publication of the case reports, four patients were alive, nine patients were dead (seven patients died shortly after diagnosis and hospitalization and two after a few months due to disease progression), and one patient was lost to follow-up.

**Table 3:** Cases of hyperthyroidism and testicular germ cell tumors published in the literature since 2000.

	Age	Clinical presentation	Tumor type and extent	β-hCG	Thyroid function testing	Clinical Course
**Chowdhury et al. (2000)** (20)	29	Weight loss, diarrhea, palpitations, sweatiness, tachycardia, goitre prominent eyes, and periorbital edema	Mixed germ cell tumor, M0	6,360 IU/L	FT4, 18.2 pmol/L; TSH, 1.1 mIU/L	Euthyroid after surgery
**Goodarzi et al. (2000)** (19)	32	Fatigue, tachycardia, and gynecomastia	Choriocarcinoma, M1	5.84 × 10^5^ IU/L	FT4, 15.5 pmol/L; TSH, <0.02 mIU/L	Multiorgan failure and death during chemotherapy
**Tilbrook et al. (2004)** (21)	58	Shortness of breath, tachycardic, and weight loss	Germ cell tumor, M1	9.95 × 10^5^ IU/L	FT4, 40.5 pmol/L; TSH, 0.01 mIU/L	Exitus for cardiac arrest despite the start of thyrostatic therapy
**Voigt et al. (2007)** (18)	27	Hematemesis, melena, anemia, and anisocoria	Non seminoma germ cell tumor, M1	>2 × 10^5^ IU/L	FT4, 35.6 pmol/L; TSH, <0.05 mIU/L	Euthyroid after chemotherapy and surgery
**Kohler et al. (2011)** (22)	31	Headache, hemoptysis, diarrhea, and vomiting	Non seminoma germ cell tumor, M1	5.54 × 10^5^ IU/L	FT4, 94.8 pmol/L; TSH, <0.01 mIU/L	Euthyroid after chemotherapy
**McCracken et al.** (2012) (23)	18	Back pain, dyspnea, weight loss, and hemoptysis	Germ cell tumor, M1	1.12 × 10^6^ IU/l	FT4, 38.6 pmol/L; TSH, <0.01 mIU/L	Exitus for respiratory failure within 24 h of starting chemotherapy
**Heda et al. (2013)** (24)	46	Abdominal pain, melena, shortness of breath, tachycardia, palpitations, weight loss, and heat intolerance	Choriocarcinoma, M1	>7.5 × 10^5^ IU/L	FT4, 29.6 pmol/L; TSH, 0.12 mIU/L	Death of disease 11 months after the start of treatment
**Arrabal-Polo et al. (2012)** (25)	33	Chest pain, palpitations, dyspnea, and weight loss	Embryonal carcinoma, M1	8.34 × 10^5^ IU/L	FT4, 99 pmol/L; TSH, 0.01 mIU/L	Exitus 24 hours after surgery for sudden severe tachycardia, tachypnea and dyspnea
**Pallais et al. (2015)** (26)	21	Fatigue, weight loss, back pain, bilateral swelling of the breast tissue, hematochezia, cough, sweats, nocturia, dark urine, and intermittent tremors	Choriocarcinoma, M1	>50 × 10^3^ IU/L	FT4, 33,5 pmol/L; TSH, <0.01 mIU/L	Improvement of clinical condition while undergoing chemotherapy
**Sotello et al. (2016)** (27)	26	Fever, chills, cough, hemoptysis, dyspnea, anxiety, palpitations, hand tremors, and weight loss	Choriocarcinoma, M1	6.16 × 10^5^ IU/L	FT4, 53 pmol/L; TSH, <0.01 mIU/L	Euthyroid after the first cycle of chemotherapy, but, then, the patient was lost to follow-up
**Shah et al. 2018** (28)	34	Painless scrotal swelling, weight loss, palpitations, diarrhea, hemoptysis, and shortness of breath	Mixed germ cell tumor, M1	8.34 × 10^5^ IU/L	FT4, 36 pmol/L; TSH, 0.01 mIU/L	Death of disease despite initial remission after surgery and chemotherapy
**Martińez et al. (2020)** (29)	17	Dyspnea, productive cough, hemoptysis palpitations, and fever	Choriocarcinoma, M1	>2 × 10^5^ IU/L	FT4, 29.6 pmol/L; TSH, 0.02 mIU/L	Antithyroid drugs and chemotherapy was started, but the patient rapidly died of respiratory failure
**Tannous et al. (2021)** (30)	29	Shortness of breath, tachycardia, and scrotal swelling	Mixed germ cell tumor, M1	1,063 IU/L	FT4, 39.9 pmol/L TSH, <0.01 mIU/L	Death of disease due to progressive disease despite chemotherapy
**Chivukula et al. (2021)** (31)	Young adult	Abdominal pain, intractable nausea, vomiting, and fever	Choriocarcinoma, M1	6.76 × 10^6^ IU/L	FT4, 64 pmol/L; TSH, <0.005 mIU/L	Despite achieving control of hyperthyroidism with chemotherapy, the patient succumbed to rapid disease progression

## Conclusion

Despite being a rare event, the presence of hyperthyroidism or thyrotoxicosis without clear etiology in a young man should lead to consider less frequent causes such as TGCTs.

Even if patients typically have mild symptoms that resolve after chemotherapy, in rare cases, a clinically significant thyrotoxicosis may arise, requiring immediate thyrostatic treatment along life-saving chemotherapy.

## Data availability statement

The original contributions presented in the study are included in the article/supplementary material. Further inquiries can be directed to the corresponding author.

## Ethics statement

Written informed consent was obtained from the individual(s) for the publication of any potentially identifiable images or data included in this article.

## Author contributions

DFa: Writing – original draft, Writing – review & editing. CO: Conceptualization, Methodology, Supervision, Writing – original draft, Writing – review & editing. CS: Conceptualization, Methodology, Supervision, Writing – original draft, Writing – review & editing. PR: Conceptualization, Methodology, Supervision, Writing – original draft, Writing – review & editing. FCa: Writing – review & editing. MC: Writing – review & editing. SR: Writing – review & editing. FG: Writing – review & editing. GR: Supervision, Writing – review & editing. DFe: Supervision, Writing – review & editing. GF: Methodology, Supervision, Writing – review & editing. FCo: Methodology, Supervision, Writing – original draft, Writing – review & editing.
